# Characterization of Plasmid pPO1 from the Hyperacidophile *Picrophilus oshimae*

**DOI:** 10.1155/2011/723604

**Published:** 2011-09-20

**Authors:** Angel Angelov, Jörn Voss, Wolfgang Liebl

**Affiliations:** ^1^Lehrstuhl für Mikrobiologie, Technische Universität München, Emil-Ramann-Straße 4, Weihenstephan, 85354 Freising, Germany; ^2^Institut für Mikrobiologie und Genetik, Georg-August-Universität Göttingen, Grisebachstrasse 8, 37077 Göttingen, Germany

## Abstract

*Picrophilus oshimae* and *Picrophilus torridus* are free-living, moderately thermophilic and acidophilic organisms from the lineage of *Euryarchaeota*. With a pH optimum of growth at pH 0.7 and the ability to even withstand molar concentrations of sulphuric acid, these organisms represent the most extreme acidophiles known. So far, nothing is known about plasmid biology in these hyperacidophiles. Also, there are no genetic tools available for this genus. We have mobilized the 7.6 Kbp plasmid from *P. oshimae* in *E. coli* by introducing origin-containing transposons and described the plasmid in terms of its nucleotide sequence, copy number in the native host, mode of replication, and transcriptional start sites of the encoded ORFs. Plasmid pPO1 may encode a restriction/modification system in addition to its replication functions. The information gained from the pPO1 plasmid may prove useful in developing a cloning system for this group of extreme acidophiles.

## 1. Introduction

*Picrophilus torridus* and *Picrophilus oshimae* are the most extreme organisms with respect to acidophilic combined with thermophilic lifestyle known to date. These species represent thermoacidophilic archaea, originally isolated from a dry solfataric field in Northern Japan [1]. Together with the genera *Thermoplasma* and *Ferroplasma* they form a phylogenetically distinct group of free-living, moderately thermophilic and acidophilic organisms within the *Euryarchaeota*. The two species of the *Picrophilus* genus are so far unsurpassed in their ability to grow at pH values around 0, with an optimum at pH 0.7. Also, *P. oshimae* has been shown to maintain an unusually low intracellular pH of 4.6, in contrast to other acidophilic organisms where this value is usually close to neutral [2]. *P. torridus* and *P. oshimae* share similar physiological properties and are morphologically indistinguishable. On the other hand, they differ in their DNA restriction fragment patterns, their 16S rDNA gene sequences, and the presence of extrachromosomal elements. Plasmids of 8.3 kb and 8.8 kb, which showed strong cross-hybridization in southern blot analysis, have been isolated from samples later assigned to *P. oshimae* but not from samples assigned to *P. torridus* [3]. Unlike the situation in the *Sulfolobales* order and especially in the genus *Sulfolobus*, where a large number of genetic elements have been characterized [4], little is known of extrachromosomal elements in the Thermoplasmatales. To date, the only sequenced and characterized plasmid from this phylogenetic order is pTA1 isolated from *Thermoplasma acidophilum* [5]. The analysis of plasmid pPO1 from *P. oshimae* reported here should prove useful in developing genetic tools for this group of organisms.

## 2. Materials and Methods

### 2.1. Strains and Plasmids

*P. oshimae* was obtained from DSMZ (DSM 9789) and was grown in a modified Brock's medium with a pH of 0.7 at 55°C as described previously [3, 6]. The *E. coli* strain JM104 (*pir^+^*) was kindly provided by Professor Ruth Schmitz-Streit (University of Kiel, Germany). pPO1 was isolated from exponentially growing *P. oshimae* cells using a QIAprep Miniprep Kit (Qiagen), and total DNA used in real time PCR was prepared by the alkaline lysis method [7].

### 2.2. Transposon Insertion and Sequencing of pPO1

Random insertions of an *E. coli* replication origin containing transposon in pPO1 were generated with the EZ-Tn5 〈R6K*γ*ori/KAN-2〉 Insertion Kit (Epicentre Biotechnologies) as described by the manufacturer. *E. coli* JM104 was transformed with 1 *μ*L of the transposition reaction and plated on LB medium supplemented with kanamycin (50 *μ*g/mL). A total of 30 plasmids were recovered from the kanamycin-resistant colonies and were sequenced bidirectionally from the ends of the Tn*5* transposons. The obtained sequences were assembled with the Staden Package software (http://staden.sourceforge.net/), and the remaining gaps were sequenced by the primer walking method. The complete plasmid sequence was determined on both strands with an average sequence coverage of 3.4.

### 2.3. Determination of pPO1 Copy Number

The copy number of pPO1 was determined with quantitative PCR (qPCR) by analysing the ratio of plasmid to genomic DNA in *P. oshimae* cells grown to the beginning of the stationary phase. Two plasmid (RT1 and RT2) and one genomic (16S rRNA gene) loci were chosen for qPCR, and the determined amplification efficiencies (*E*) were *E*_RT1_ = 1.58 (*R^2^* = 0.998), *E*_RT2_ = 1.95 (*R^2^* = 0.997), and *E*_16S_ = 1.62 (*R^2^* = 0.993). The real-time PCR measurements were carried out in triplicate with two independent preparations of total *P. oshimae* DNA which permitted the estimation of the intra- (C_intra_) and interassay (C_inter_) coefficients of variation, C_intra_ = 1.32% and C_inter_ = 2.02%. These assays were performed using SYBR green (qPCR MasterMix Plus for SYBR green I with fluorescein, Eurogentec) on an iCycler (Bio-Rad); the primers used are listed in Table 1. 

### 2.4. Sequence and Transcriptional Start Sites Analyses

ORF prediction was performed on the EasyGene prediction server (http://www.cbs.dtu.dk/services/EasyGene/); the nucleotide and protein sequences and conserved domains were searched with the NCBI database using the programs BLAST and CDART, respectively. Repeats were identified with the Tandem Repeats Finder program [8]. In order to determine the transcription start sites of the pPO1 ORFs, RNA ligase-mediated rapid amplification of cDNA ends (RLM-RACE) was used [9], using the protocol of the manufacturer (FirstChoice RLM-RACE kit; Ambion). Two independent replications of the 5'-RLM-RACE procedure were carried out, including a control without tobacco acid pyrophosphatase treatment. The cDNA obtained was subjected to nested PCR with SuperTaq Plus DNA polymerase (Ambion) using the primers listed in Table 1; the obtained PCR products were column purified (QIAquick PCR purification; QIAGEN) and cloned using a StrataClone PCR cloning kit (Stratagene). A total of 10 colonies per ORF were analyzed, and the plasmid inserts were sequenced in both directions with an ABI 3700 sequencer (Applied Biosystems).

## 3. Results and Discussion

### 3.1. Structural Features and Open Reading Frames in pPO1

**Figure 1:**
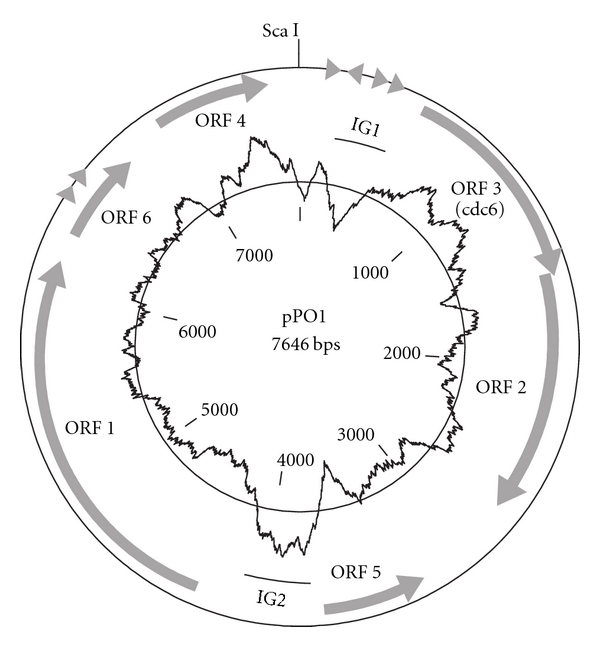
Schematic map of the pPO1 plasmid. Arrows designate ORFs, repeats are marked as triangles, and deviation from the average GC-content is plotted on the inner circle.

Complete sequencing of the plasmid pPO1 from *P. oshimae* resulted in a circular molecule of 7646 bp (GenBank accession number JN032732) and a G + C content of 30.5%. This is considerably lower than the G + C content value of 36% reported for the genomes of *P. oshimae* (HPLC data, [3]) as well as for *P. torridus* (genome sequencing data, [10]). Two oppositely situated intergenic regions in pPO1, IG1 and IG2, were found to deviate significantly from the average G + C content (Figure 1). Interestingly, no Tn*5* transposon insertions were found in these regions among the 30 plasmids used for sequencing. A possible explanation for the observed bias could be the formation of stable secondary DNA structures rather than the local G + C content which can prevent Tn*5* transposition [11]. The intergenic region IG1 was also found to be rich in repeated sequences. A total of four tandem repeats were detected, two of which were localised in IG1 (Table 2).

The nucleotide sequence displayed no regions of homology to other archaeal plasmids or to the genome of *P. torridus*. A total of 6 ORFs longer than 100 amino acids could be predicted from the nucleotide sequence. ORFs 1 to 4 contained domains which could be assigned to known COG and/or PFAM families (Table 4). ORF 3 appeared to encode a protein of 42.4 kDa which showed the sequence characteristics of an archaeal Orc1/Cdc6 cell division control protein and displayed a high level of amino acid sequence similarity (38% identity) to the Orc1/Cdc6 homologue found on the pTA1 plasmid from *T. acidophilum* [5]. Although Rep proteins are most often implicated as replicator initiators of plasmids, no homolog of a known Rep protein could be identified in pPO1. We could not detect single-stranded DNA intermediates in cell extracts of exponentially growing *P. oshimae*, neither by nondenaturing southern hybridization nor by S1 nuclease-based protection assay [12] (data not shown). Therefore, most probably pPO1 replicates via a theta mechanism, and ORF 3 is likely to be a replication initiator. The localization of ORF3 and the intergenic region IG1 further supports this prediction as IG1 carries the characteristics of an archaeal replication origin site (*oriC*). Archaeal *oriC* sites are typically AT-rich, repeat-containing intergenic regions and are most often located upstream of genes coding for Orc1/Cdc6 homologs [13]. Similar overall architecture of the Orc1/Cdc6 ORF and the oriC site can be found in the pTA1 plasmid from the related *T. acidophilum* [5]. Clearly, further experiments are needed to unequivocally determine the mode and origin of replication for both pPO1 and pTA1. 

Additionally, pPO1 seemed to encode genes for a restriction/modification system (R/M system, ORFs 5 and 2) and for a recombinase (ORF 4). Notably, the ORFs of the putative R/M system were found to have homologs only in the bacterial lineage, the ones with the highest level of amino acid sequence similarity belonging to the group of high GC Gram positive Bacteria.

### 3.2. Plasmid Copy Number Determination

pPO1 copy number was determined by measuring the abundance of two plasmid DNA loci relative to a genomic DNA locus using quantitative real-time PCR. The results obtained for the two plasmid loci were in good agreement with each other, that is, 11.8 ± 3.6 copies per genome equivalent for RT1 and 15.4 copies ± 2.7 for RT2. The DNA samples used for these measurements were from cultures grown to the beginning of the stationary phase.

### 3.3. RACE Analysis of Transcriptional Start Sites

The transcriptional start sites (TSS) of four of the pPO1 ORFs could be mapped by the RLM-RACE procedure (Table 3). No PCR products were obtained for ORFs 3 and 4, most probably indicating low levels of transcription of these ORFs under the experimental growth conditions. Despite the colinear organisation of ORFs 1, 6, and 4 and in addition the overlap of ORFs 3 and 2, no polycistronic RNA could be detected by 5′ RLM-RACE.

### 3.4. Potential of pPO1 for Vector Development

To date no cloning system or transformation method is available for the hyperacidophilic organisms of the *Picrophilus *genus. Plasmid pPO1 could now serve as the starting point for development of a recombinant cloning vector. Judging from the pPO1 sequence, the size of the plasmid regions needed for autonomous replication to be included in the construction of a shuttle vector can presumably be reduced considerably, for example, by deletion of the R/M system genes. A host/vector system for the genetic modification of *Picrophilus* strains will help to study in more detail the basis of extreme thermoacidophilic adaptation in these unique archaeal microorganisms which are capable of life around pH 0.

## Figures and Tables

**Table 1:** Oligonucleotides used in this study.

Primer	Sequence (5′–3′)	Description
RT1.for/rev	AATATGCCCTGGAGATAGCG/AGACGACACTTTCCGATACG	Used in quantitative PCR for plasmid copy number determination
RT2.for/rev	ATGCACGAATGCCTACTCTC/GGGCAAAGGAAGGCTTAAAC	
16S-Pt.for/rev	TCGTCCTTCCAGGATTACAG/CCGGCTTTGTAAATCTCCAG	
RC1.rev1/2	TCGGATTTAACAGGGCGTCTA/CCTGAACTGCCTGTTATCAAT	ORF1 inner/outer 5′RACE
RC2.rev1/2	CAATAGGGTGGAGATGTTATAGC/TCAAAGCCCAGCCATTAGATTGC	ORF2 inner/outer 5′RACE
RC3.rev1/2	TTACCTGACCCTTTATTTCC/TCCAGGGCGTTTAAGTAAATG	ORF3 inner/outer 5′RACE
RC4.rev1/2	TTTCCCTGATATTCCCTTATCC/ACATCTCTTGGTATGCCTTTC	ORF4 inner/outer 5′RACE
RC5.rev1/2	TCGGAGTAGAATTTACCTGTAG/GGATAAGGATTACCTCTGTTAG	ORF5 inner/outer 5′RACE
RC6.rev1/2	GCATTTAGGACAGGTCGCATAC/ACTCACTACCACTTACATAC	ORF6 inner/outer 5′RACE

**Table 2:** Tandem repeats in pPO1.

Tandem repeat	Consensus sequence	Coordinates	Period size	Copy number
TR1	TTAATTAAAAAAAATAAAAAATAT	283–328	24	2.0
TR2	ATAATATAATATA	384–431	11	3.8
TR3	AAGTAATGGTAAGTAAAGATGT	6382–6431	21	2.3
TR4	AAAAGTAAGTAAGAAAGTAAGTAATAGTGAGTTAAAACCTC	6452–6530	42	1.9

**Table 3:** Transcriptional start sites (TSS) of pPO1. ORFs identified by 5′RLM-RACE. The start codons of the ORFs are in bold, the TSS are preceded by an asterisk.

ORF-1	ORF-2
…ATCAATTATTCTCTGTTTTACTTTT∗ACTATTTTATTTTACTCATTATAATAATTAGGTTAATAGTATATATCTATAGACGCCCTGTTAAATCCGAGTTT**ATG**CAGACTAATTACATCAC…	…TAAAGGCATTGATGAAA∗GAACCTAATACAAACAAGGCTTACACAATCACAGAAAATTATATGAATGAAATTTTAGGATATTCAATAAAGAAAAGGCAGTATTTTAACATCATACACGACCTAAATAATTTAGGTTTATTCAACATCGTGAGAAAAAGAGAAGGCAGGTATTACACCCTTGAAGTGCAAATGCTAATGCCAGACAACAAGTTAATTCAAAATGAATTAAAGAACAGGTTTAATATAA**ATG**GGTGATAA…

ORF-5	ORF-6

…TATGACAAAGCATCAGAATTAGATGT∗GGGGCAGAAAGGGTATTTCATAATCTTTTTGGAAGGTTTCAA**ATG**CTACAGGTAAATTCTACT…	…CATTAAGGGAATTGCAAGAGATGAAA∗GATACAGAGGAATATAAGTAAGTGGTAAGTAGTAAGTAAGTAGATAAGCTTATTAACTTACTCACTTATACTATTATT**ATG**ATATTAACATGC…

**Table 4:** Annotation of the predicted ORFs of pPO1 and assignment to existing COGs.

ORF	COG family (description)	Annotation
1	COG0433, (FtzK, DNA segregation ATPase FtsK)	FtzK-related protein
2	COG0863, (DNA modification methylase)	Putative N6–N4 Mtase
3	COG1474, (Cdc6-related protein, AAA superfamily ATPase)	Cdc6
4	COG1961, (PinR, site-specific recombinases)	DNA invertase/resolvase family protein
5	No	Type II restriction enzyme
6	No	Beta-subunit of acetyl-CoA carboxylase
